# Towards the development of a reflective radiographer: challenges and constraints

**DOI:** 10.2349/biij.4.1.e9

**Published:** 2008-01-01

**Authors:** MA Baird

**Affiliations:** Department of Medical Imaging and Radiation Sciences, Faculty of Medicine, Nursing and Health Sciences, Monash University, Melbourne, Australia

**Keywords:** Education, reflective practice, curriculum

## Abstract

Currently there is overwhelming support from the health professions for universities to devise curricular approaches that lead to the development of undergraduate reflective skills, and over time, reflective practice. However, in the case of radiography, irrespective of the degree to which radiographers might engage in reflective practice they constantly struggle to shed the perception they are little more than technical operatives. The four-year Bachelor of Radiography and Medical Imaging was introduced by Monash University in 1998 with an overt commitment to the development of a reflective radiographer. Findings from student and supervisor surveys generally support the program and its aims. However, as the findings and student feedback will attest, many challenges and constraints continue to face educators who seek to situate their curriculum within the reflective practice paradigm.

## INTRODUCTION

Preparing students for entry into the health profession has always been a challenging undertaking. The curriculum must not only keep up with the relentless developments in biomedical knowledge, technology and engineering it must also pay serious attention to what is now known about the development of clinical expertise [1]. At the same time, health professionals are being increasingly mandated by Registration Boards and Government and professional bodies to actively apply critical thinking within the workplace, demonstrate reflective, creative, communicative and interpersonal skills and, by association, engage in “reflective practice” [2-8]. Therefore, it is no longer satisfactory for educators within the health profession to simply develop the knowledge base of their students. Educators are increasingly obliged to ensure their students develop the kind of personal and intellectual capacities that will lead to defensible and ethical decision-making that is grounded in the best available “evidence” [9].

Indeed, there has been a paradigm shift towards the need for educators to close the “gap” between the seemingly stable world of the academic-shaped as it is by objective and generalisable scientific theories and the somewhat chaotic world of the practitioner in which experiential knowledge is accorded a privileged position [1, 10-14]. Additionally, within radiography at least, educators must respond to pressures from the profession for graduates who are technically competent [15-16].

As radiography was the last of the health professions to upgrade from diploma to a degree level of education [17-18], it is not surprising that in keeping with the trends of the 1990s, academics followed the lead established initially within teaching and, then nursing, by embracing the reflective practice paradigm as a means of bridging the so-called “gap” between academia and the practice setting [19-22]. However, questions are now being raised within nursing as to whether engagement with the reflective practice paradigm is worth the effort [2, 6].

This paper will demonstrate how the Bachelor of Radiography and Medical Imaging has been structured to meet the new challenges facing radiography educators. In the process, the paper will argue that educators must continue with their efforts to expose students to the principles associated with the reflective practice paradigm. At the same time, given the technological and socio-political context within which radiography is practised, and the nature of clinical expertise, it will be argued that educators need to place the practice of radiography at the heart of the curriculum and ensure the academic content of their courses is contextualised to the needs and concerns of the practice setting.

## THE VALUE OF THE REFLECTIVE PRACTICE PARADIGM IN INFORMING THE CURRICULUM*

Why should educators embrace the reflective practice paradigm? Because engagement in reflective thinking is how practitioners create knowledge as individuals and, as members of a “community” of practitioners [24-25]. Reflective thinking is also the bulwark against the all too easy slide into “habitual” and, in extreme cases “dysfunctional” practice [26-28].

*A recent critical clinical learning experience from a 2007 first year Monash radiography student (extracts reproduced with permission – Please note: all student and practitioner names including hospitals are pseudonyms) is instructive in this respect:

“The hospital I went had a vast range of patients and poses a great threat to the spread of infection (sic)… An elderly inpatient was brought to the radiology department from a ward for abdominal x-rays. The ward was known to have patients with gastro. In general most of the radiographers involved in the examination used protective measures… There was however a radiographer … who found it unnecessary to apply protective barriers and follow the hospital protocol… I found the situation troublesome. I believe the situation was handled poorly by that specific radiographer and perhaps even by other radiographers involved. None of the radiographers who correctly followed the infection control protocol took it upon themselves to check everyone participating in the examination was wearing protective clothing before the patient was handled… Ultimately, I believe the level of patient care was compromised due to the failure of a radiographer to correctly follow precautionary measures”.

Educators have always known that the learning of theory cannot tell someone how to practise [25, 29]. Neither do competency-based descriptions of professional practice adequately capture the essence of professional knowledge [2, 23, 30 - 32]. Even in a profession that is completely mediated by technology such as radiography, Schön’s [23] assertion that traditional forms of knowledge are bounded in the practice setting by “artistry” in the form of “problem framing”, “improvisation” and “implementation”, holds true. The following excerpt from a 2000 first year Monash radiography student’s (extract reproduced with permission) critical clinical learning report illustrates this point:

“By the end of the first week, it was clear that not all doctor’s requests were precisely correct in terms of the radiographs required. Often, much more information is relayed directly from verbal communication with the patient. One such outstanding case appeared whilst I was assisting Kathy (a radiographer with 8 years experience) on an in patient (sic). The request form for this elderly patient had ordered for an AP and lateral projection of the hip joint (normal protocol in the hospital) as she had undergone a total hip replacement. However, the patient continually complained about her leg. After querying about where and how the pain was initiated, it was decided that a shot of the whole femur would be done. The commendable outcome was that a fracture at the tip of the pin was captured – and only seen on the AP projection (sic). If the conventional projections suggested by the doctor had been undertaken, such a fracture would have never been seen. Due to rational communication with the patient the problem was identified”.

Therefore, whilst students need to know the rules and underlying principles of the practice, they need to be given opportunities to learn how to adapt these rules and fit the theory to the particular problem at hand [23, 33-34]. According to Harris [35], it was Schön who demonstrated that competent practical action is contingent upon competent practical reasoning which involves “knowing-in-action”, “reflection-in-action” and “reflection-about-action”. Furthermore, it is the ability of the practitioner to define the problem or make a puzzling situation coherent, which Schön [23] argues is the crucial precursor for the application of technical or scientific knowledge to the problem at hand.

Reflective practitioners are unusually adept at handling situations of uncertainty, uniqueness and conflict [24]. In the midst of confusion, they bring to the surface a host of cognitive and emotional assumptions, critically re-examine them, pose new questions, construct effective judgements and, in the process, develop new or modified action theories [5, 23, 29, 36-37]. Reflective practitioners know how to use reflective processes to break out of habitual responses to their practice [38].

## IS THE REFLECTIVE PRACTICE PARADIGM STRONG ENOUGH TO OVERCOME THE INFLUENCE OF THE PREVAILING SOCIO-POLITICAL CONTEXT?

Few educators would disagree that reflective practice is a “good thing” [39]. Nevertheless, revelations from patients, whistleblowers and students raise the question as to whether the reflective practice paradigm is robust enough to truly prepare students for entry into professions that are now simply seen as part of the health care industry, which is increasingly dominated by the “invisible” hand of the marketplace and recourse by patients to litigation [40-41].

For example, it appears that the public hospital system in Australia remains in a permanent state of “chaos” [42-44]. Many in the wider community must be wondering how members of professions supposedly dedicated to meeting the needs of the sick and dying, could systematically ignore the pleas of a woman in a major Sydney emergency department before she miscarried in the toilet. The woman’s husband reportedly criticised the hospital for providing no “care and comfort” [45]. The following extract from a student informant who was given the name Veronica, gathered in the context of the author’s doctoral study into the practice of radiography supports this experience of public hospital care:

“I was questioned by my supervising radiographer as to what I was doing? I said that I was helping the patient change as the patient was having difficulties. Also, because we were not busy, I felt that I had time to chat to the patient. I was told not to waste my time talking to my patients. My supervisor told me if they want better service they should go to a private practice’ (Baird, 1998, p337).

Unlike other health professions, radiographic practice is completely mediated by technology. Irrespective of the imaging modality, radiographic practice in Australia is also supervised by medical practitioners many of whom are partners within a profit-driven radiology industry [41, 47-48]. As a consequence, attempts by radiographers to engage in “virtuous” practice are regularly influenced by the imaging protocols established by radiologists and the imperatives of the sector to meet its “bottom line” [41, 48].

Sonia (another informant) found the habit of radiographers in “hurrying” patients along instead of stopping and explaining things to them, quite disconcerting. She told me that the radiographers she had worked with “get the patient against the bucky with the cassette in place before the patient has had a chance to comprehend what is going on”. In fact, Sonia found that during her time at Primrose Square Hospital the radiographers made her feel that she had been in the way. Their overriding concern was “to get the patient done and get on to the next one” (Baird, 1998, p334).

These examples confirm the observations made by Eraut [28] - when he argued that due to institutional pressures and constraints, the critical monitoring of practical action can become difficult to maintain. As a consequence, mistakes occur and patient care is compromised. The following excerpt from a 2002 first year Monash radiography student’s critical clinical learning report (extract reproduced with permission) provides an illustration of what many would regard as poor professional practice:

“The initial breakdown in communication was not helped when the attending doctor told the patient in much the way as referring to a mischievous child “you have two choices you either have the x-rays or you go out of the door!” The radiographer then proceeded to use forceful actions to move the patient … most communication was blunt”

According to the student, the patient in question was not under the influence of any drugs. Pressures to conform to “entrenched custom and practice” [49] within the health sector characterises the delivery of health mitigating against effective service delivery virtually forcing busy practitioners to develop habitual approaches to their practice [40, 46, 49].

“The communication element of the Professional Skills subject was good but not realistic. You don’t have time to sit down with the patient for 5 minutes beforehand for a chat” (Monash Student 22, clinical debriefing session Semester 1 Year 1 2000)

Hence, it should not come as a surprise that at least within nursing, concerns are now being expressed as to whether the “certitude” surrounding the capacity of the reflective practice paradigm to produce “better patient care” is justified [2, 6].

However, educators must not give up. There are very valid reasons why the reflective practice paradigm should command our attention. Dewey [26] argued a long time ago that it is only through reflective thought that individuals can be empowered to take control of their destiny. It is only through a conscious engagement in reflective thinking, he believed, practitioners could more fully know themselves and, in the process, come to adopt an inquiry-based approach to their actions [50]. However, at the same time, Dewey also recognised that those human capacities of flexibility, responsiveness and self-awareness need to be developed and that the level of their development is contingent upon the kind of experiences students have during their undergraduate years [26, 50-53].

## RESPONDING TO THE CHALLENGES

As the previous discussion has plainly indicated, educators face a daunting task in developing a curriculum that is strong enough to prevent it from simply reinforcing the “reproduction” of radiography in the way it has always been performed. Whilst students are provided with lectures, tutorials, laboratory exercises, positioning classes and multi-media programs about radiography, it is only when they actually experience what it is like to position a patient for a radiograph that the potential for meaningful entry into the radiography profession can be realised.

“The clinical tutor did an excellent job in assisting me. He provided almost constant supervision without being smothering. This has definitely resulted in a highly beneficial experience” (Monash radiography student, Year Two Semester One, 2001)

Hence, the curriculum must address the role played by experience in creating the conditions for learning to occur and the role of reflection in creating knowledge. Secondly, the curriculum must ensure students develop an appropriate knowledge base and realise the value of propositional knowledge in the workplace. In other words, educators must address the “interplay between formal knowledge … and experiential knowledge” in the development of clinical expertise [1]. Thirdly, there is a need for educators to use a model of clinical skill development that acknowledges the developmental nature of skill development and the complexity of professional competency whilst at the same time reminding practitioners that judgments about a student's performance must reflect the experiential level of the student. Finally, strategies must be in place to support students as they confront the reality of front line radiographic practice.

### The philosophical position of the Bachelor of Radiography and Medical Imaging

The four-year Bachelor of Radiography and Medical Imaging commenced in 1998. The curriculum was heavily influenced by the research findings of the author. In essence, as a consequence of an extended period of data collection in the “field” utilising participant methodology, the author came to the conclusion that existing curricular approaches failed to empower graduates to change radiographic practice [46].

Thus, the Monash course reflects a strong commitment to the experiential learning theory first espoused by Dewey and his notion of reflective thinking later re-defined by theorists such as Kolb [54] in terms of a learning cycle and Schön in terms of reflective practice [24]. It acknowledges the contextual learning theory espoused by Coles [55] and, just as importantly, mental schema theory from cognitive psychology [56-57]. Attention is also paid to the insights educators have gained from the critical theory associated with Habermas [58] and constructivism with its origins attributed to Kant, Piaget and the pragmatic school of philosophy [59-60]. The clinical assessment tools used within the clinical studies units are congruent with the “novice to expert” model of skill development associated with Dreyfus and Dreyfus [61] and introduced into nursing by Patricia Benner.

## PHASE ONE: TRANSLATING THE PHILOSOPHY INTO A PRACTICAL REALITY

If the gap between the university and the clinical world is to be bridged, it is helpful to conceptualise the curriculum in terms of a learning campaign with distinct yet interrelated phases [62]. Phase one relates to the on-campus program.

The contextual learning theory associated with the work of Coles [55] originally informed the creation of a vertically and horizontally integrated academic program. It was Coles [55] who demonstrated that without a clear clinical professional context being presented to students, theory remains inert and is treated by students as somewhat superfluous to practice. Therefore in the Monash course, students are introduced to anatomy, physiology, pathology, and radiographic anatomy and pathology a semester at a time so a strong alignment with the corresponding imaging modality, which is addressed in either the Radiographic Imaging and Methods or Medical Imaging and Methods units and experienced in the clinical rotation, can be achieved. In the first year of the course this means general radiography and in the second year complex general radiography and digital subtraction angiography (DSA). In the third year, students engage in computed tomography (CT) and medical ultrasound (US). In final year, students study magnetic resonance imaging (MRI), undertake additional CT and DSA clinical studies and complete an inquiry-based research project.

In the first year Radiographic Imaging and Methods units and in the third year Medical Imaging and Methods units students also actively engage in a series of practical classes in general radiography, medical ultrasound and CT. These classes are designed to facilitate the development of relevant schemata without which students struggle to structure and make sense of their clinical experiences.

Contextual learning theory makes other demands upon educators. Attention must also be paid to the teaching methods used within the course. From the second year onwards, a case-based learning with a computer-based program, developed within the Department by members of the course team, is increasingly used. It is called Student Oriented Learning About Radiography and referred to as SOLAR. This teaching approach additionally promotes the use of group learning activities and, through the medium of case presentations, peer learning [63].

### The development of professional skills

There are four professional skills units within the course. It is these units that prepare students to complete the second type of Clinical Learning Contract used during clinical studies, namely the Professional Development Learning Contract.

In the first year, radiation safety and ethics, the concept of profession, infection control, professional communication, first aid, principles of scientific writing and the psychosocial basis of behaviour and illness are addressed. The link between these topics and the clinical world is achieved by students through the completion of a structured observation of clinical communication skills and critical clinical professional learning experience reports using a style reported by Benner [64]:

- Introduction: what happened
- How the experience affected my professional development
- My thoughts about the situation
- What the theory has to say about the situation
- The significance of the experience
- Conclusion

The second semester in second year professional skills unit is concerned with facilitating knowledge about patient assessment and management from a nursing perspective, the evidence-based paradigm, radiation therapy, nuclear medicine and research methodology. The final professional skills unit is embedded within the second semester in the third year unit dealing with breast imaging and dosimetry where students undertake additional study in ethical theory and medical law. Students apply this new knowledge during their clinical placement by completing a series of critically reflective learning reports.

## THE SECOND PHASE: THE MANAGEMENT OF THE CLINICAL STUDIES UNITS

The management of the clinical studies units, of which there are eight, is highly structured and characterised by the use of Learning Contracts, Learning Portfolios, and the Novice to Expert Model of Clinical Skill Development.

### Learning contracts

Throughout their clinical studies’ attachments, students use learning contracts as a means of ensuring clarity about the objectives of each clinical rotation and the nature of assessment. The supervising radiographer and student discuss and sign the contracts. The signature of the student indicates their intention to do their best to complete the contract. To record progress in meeting Learning Contracts for the Development of Competence in Radiography, space is provided within the various Workbooks for students to write up Weekly Case Reports and Log Entries. The nature of these reports and an analysis of the structure of the Clinical Workbooks will be the subject of another article.

### Assessment strategies within clinical studies

All of the clinical assessments are structured around the idea that students will move through the various stages from beginner to intermediate beginner and advanced beginner practitioner and finally attain the level of competent practitioner by the end of the course. By the end of the first year, students demonstrate the characteristics of an advanced beginner in relation to general radiography performed on cooperative patients. By the end of the second year, they achieve the advanced beginner stage in respect to contrast imaging, mobile radiography and trauma imaging. The level of competent practitioner in general radiography is achieved by the end of third year. In respect to CT, students are expected to reach the level of advanced beginner by the end of the third year and the status of competent radiographer by the end of the fourth year. The level of intermediate beginner in ultrasound of the upper abdomen is expected to be achieved by the end of third, and in MRI, in final year.

In the diploma courses in radiography, competency was seen as a linear process that could be objectively assessed. However, does the successful performance of a series of radiographic steps constitute competence? At degree level of education, students must know how to perform radiographic examinations and be able to justify their clinical decisions. Indeed, in a study undertaken to ascertain if graduate nurses were better decision makers than non graduate nurses, Girot [65] concluded that “those exposed to academia are more effective decision-makers than non-graduates in practice”. It was the university experience that created the conditions for the development of critical reasoning capacities and which subsequently made the difference in the clinical setting. The same must occur for radiography students if the attainment of graduate status for the profession is to be justified.

This means clinical assessment proformas must go beyond requiring clinical assessors to make inferences from a series of observations that students know what they are doing. The proformas need to require students to verbalise their clinical decisions and actions.

The assessment strategy must also oblige students to engage in a conversation with themselves about their professional progress along the novice to expert continuum. In all year levels, students are required to complete a series of critical self assessment exercises that must be discussed with their supervising radiographers. Students must identify their strengths and weaknesses and develop personal action plans that are verified and linked to their written statements.

## THE THIRD PHASE: CLOSING THE LOOP

The final phase relates to how links with the students are maintained during the clinical studies and the need to debrief students upon their return to the university. A structured form of clinical liaison occurs during all of the clinical placements together with personal site visits and follow-up telephone contacts. Students need to feel supported throughout their clinical studies. Indeed, it is only by engaging with students and radiographers in “the field” that educators can truly evaluate the efficacy of the tools that have been created to facilitate the achievement of the clinical studies objectives.

## IS THERE ANY EVIDENCE THAT MONASH GRADUATES ARE DEVELOPING INTO REFLECTIVE PRACTITIONERS?

The results from two surveys conducted in 2001 and 2006, respectively, appear to provide some evidence that the majority of graduates from the Bachelor of Radiography and Medical Imaging are developing the capacity to engage in reflective practice.

### The Value of the Collection and Evaluation of Reject Radiographs in Encouraging Students to Reflect upon their Learning

In 2001, 36 students comprising the third year cohort were invited to participate in a two-week audit of their reject radiographs. Students were also asked to complete an audit form and submit a reflective piece about the value of the exercise. Students were also asked to report upon the number of sub optimal radiographs that were put through for reporting.

Twenty-two students, each of whom attended a different hospital-based radiology department or private radiology practice, submitted their radiographs. The number of rejects ranged from 3 to 120. The average repeat number per student (excluding the two extremes) was 14. The numbers, however, are not the key issue. The key research interest was the views of students regarding the usefulness of the exercise. Without exception, the students who completed the exercise found it worthwhile. The following reflective comments, firstly from Stan and secondly from Igor, gives educators cause for hope:

“Overall I found this exercise to be beneficial in making me think about why I was repeating a radiograph rather then just chucking the reject in the bin and having another shot. Hopefully being aware of why I was repeating films will mean I repeat less radiographs and will make me a better radiographer”.

“I found my collection of repeat radiographs to be quite minimal. I believe this was partly due to the fact that at my centre the staff were very helpful and friendly which made me feel comfortable enough to ask questions and perform examinations alone. In feeling comfortable I also believe that I felt more competent and as a result fewer repeats occurred. …. I am not sure as to why I had fewer repeats (being conscious of the audit or feeling more competent in performing examinations) however I was constantly thinking as to how to get the best exposure, positioning etc as to reduce my repeats. In sum I believe that the audit did get me thinking more about the examination as to reduce the number of repeats however, I also felt more comfortable and confident which I am sure also was a contributing factor as to why there were fewer repeats”.

As a result of the feedback from all of the students including the following comments from Pete, all students are now given opportunities to complete a reject analysis in the context of their Professional Development Learning Contracts in the second semester of first year and the first semester of second and third years.

“From the limited number of repeats I have had to repeat it would seem that my positioning is the largest cause of repeats and I will take more time when positioning patients to make sure this is accurate. I believe this exercise is a good reflective piece and is of benefit to myself and should be included in the second and third year of the course”.

### A 2006 audit of the quality of Monash Radiography graduates

In semester one of 2006, a formal audit of the quality of graduates from the course was conducted by the Department of Medical Imaging and Radiation Sciences. The findings formed part of the re-accreditation portfolio submitted by the Department to the Australian Institute of Radiography in July 2006. Fifty accredited clinical departments were invited to complete the questionnaire comprising 71 questions. Forty completed questionnaires were returned. The audit process was conducted by the Monash University Centre for Higher Education Quality. For the purposes of this discussion only, responses to a select number of statements have been provided below in (Table 1). Whilst many respondents ticked the 'sometimes' option, no one disagreed with any of the statements. The responses indicate that the curriculum for the Bachelor of Radiography and Medical Imaging has the potential to meet its goals.

**Table 1:** Selected responses to the audit.

**Statement Number**	**Statement**	**Strongly Agree**	**Agree**	**Sometimes**
40	Graduates display an ability to collaborate effectively with colleagues for the benefit of patients	5	20	3
44	Graduates possess the capacity to formulate innovative solutions to radiographic challenges whilst remaining sensitive to ethical, social and cultural practices	5	20	15
45	Graduates possess the ability to apply critical thinking in the workplace	7	19	14
48	Graduates exhibit a reflective stance to their work	8	19	13
49	Graduates display appropriate levels of care when interacting with patients	15	22	3
50	Graduates accord patients dignity and respect	19	19	2
61	Graduates can assess their work in a manner that indicates ongoing professional development	10	27	2

## CONCLUSION

In 1998, the author discovered a gap between the day-to-day practice of radiography and what it could be [46]. Reports from current students suggest that there are still radiographers who are failing to live up to the highest ideals associated with a profession. Therefore, irrespective of the need for radiography students to master their technologically-mediated craft, educators must continuously create learning experiences that signal to students they must come to understand that in the university setting, knowledge is something that is open to “contestation” and debate.

From within the reflective practice paradigm, knowledge is not something that is “out there” waiting to be discovered. Rather, knowledge construction occurs as students struggle to reconcile the theories and concepts found in text books with clinical realities and practical experiences. Ultimately, it is an engagement with the process of reflection that makes the creation and integration of knowledge possible and opens the door for the development of a reflective radiographer.

Thus, the challenge for radiography educators is to construct a curriculum that acknowledges the continued need for students to gain technical competency whilst attending to the university imperative that students develop those critical and reflective thinking skills traditionally associated with a “higher education” [58]. The overriding concern of the curriculum must be to empower students to develop their own critically-informed practice “scripts” [5]. Even in these difficult times of a deliberate intrusion into health care of corporatisation, manageralism and the market place [49], educators must not lose sight of the potential for education to effect change [58].
